# Facet‐Dependent Cold Welding of Au Nanorods Revealed by Liquid Cell Transmission Electron Microscopy

**DOI:** 10.1002/advs.202412779

**Published:** 2025-01-31

**Authors:** Wen Wang, Dongxing Song, Fangjie Meng, Sufeng Fan, Ran Cai, Shaobo Cheng, Chongxin Shan, Tao Xu, Haimei Zheng, Litao Sun

**Affiliations:** ^1^ Henan Key Laboratory of Diamond Optoelectronic Materials and Devices Key Laboratory of Materials Physics Ministry of Education, and School of Physics Zhengzhou University Zhengzhou 450052 China; ^2^ Engineering Technology Research Center of Henan Province for MEMS Manufacturing and Applications School of Mechanics and Safety Engineering Zhengzhou University Zhengzhou 450001 China; ^3^ School of Medical Technology Beijing Institute of Technology Beijing 100081 China; ^4^ SEU‐FEI Nano‐Pico Center Key Lab of MEMS of Ministry of Education Southeast University Nanjing 210096 China; ^5^ Materials Sciences Division Lawrence Berkeley National Laboratory Berkeley CA 94720 USA

**Keywords:** Au nanorods, cold welding, in situ TEM, welded interface, welding mechanism

## Abstract

Cold welding of metals at the nanoscale has been demonstrated to play a significant role in bottom‐up manufacturing and self‐healing processes of nanostructures and nanodevices. However, the welding mechanism at the nanoscale is not well understood. In this study, a comprehensive demonstration of the cold welding process of gold nanorods with different modes is presented through in situ liquid cell transmission electron microscopy. The experimental results and molecular dynamics simulations reveal that the nanorods are welded through the facet‐dependent atomic surface diffusion and rearrangement along {100} facets. The density functional theory calculations indicate that the preferred coalescence of two {100} surfaces is thermodynamically favorable. Unlike the prevalent “oriented attachment” in the nanoparticle coalescence, the misalignment of nanorod orientations and local stresses can induce grain boundaries and stacking faults in the welded interface.

## Graphical abstract

The cold welding dynamics of gold nanorods with different modes are invested through in situ liquid cell transmission electron microscopy. Unlike the prevalent “oriented attachment” in the nanoparticle coalescence, the experimental results and molecular dynamics simulations reveal that the nanorods are welded through the facet‐dependent atomic surface diffusion along the {100} facets.

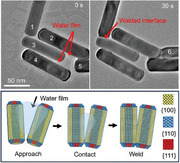

## 1 Introduction

Welding, also known as joining, soldering, or bonding, has been an ancient and important manufacturing technique since the Bronze Age.^[^ ^1^, ^2^ ^]^ Nowadays, with the advancement of nanoscience and nanofabrication, welding hits the spot in the bottom‐up fabrication of nanostructures and nanodevices. It is capable of synthesizing nanoparticles to create 1D, 2D, or 3D functional nanostructures as well as connecting nanostructures to fabricate integrated circuits.^[^ ^3^, ^4^ ^]^ Many approaches have been applied to joining the low‐dimensional nanostructures, such as voltage/current excitation,^[^ ^5^ ^]^ thermal sintering,^[^ ^6^ ^]^ electron or laser irradiation,^[^ ^7^, ^8^ ^]^ plasmon interactions.^[^ ^9^, ^10^ ^]^ Although these methods could realize the welding process, they always induce the heating effects. Since the nanomaterials are sensitive to heat due to their small sizes, local heating can be challenging to control precisely and may result in the breaking up or other fatal damage to the nanostructures.^[^ ^11^ ^]^

In recent years, cold welding which is defined as a solid‐state welding process without fusion or heating at the interface of the two parts to be welded, has garnered increased attention in nanoscience due to its minimal impact on the sample and the simple operation.^[^ ^12^, ^13^, ^14^, ^15^ ^]^ It is particularly well‐suited for constructing nanostructures and facilitating self‐healing. Currently, the nano welding technique for bottom‐up manufacturing is still in its early stages of development and is gaining momentum. Understanding the welding mechanism at the nanoscale is crucial for achieving controllable and repeatable welding, thereby expanding the scope of welding applications. Besides, the welded interface may exhibit novel properties such as deformation, grain boundaries, lattice distortion, etc., which ultimately determine the mechanical, electrical, and optical properties of the welded nanomaterials.^[^ ^16^, ^17^ ^]^ However, the welding mechanism and the structure of the welded interface remain elusive due to the lack of in situ high‐resolution characterization of the welded interface.

Cold welding can be realized in a vacuum or solution.^[^ ^3^ ^]^ Liquid cell transmission electron microscopy (TEM) can monitor the dynamic behavior of nanomaterials in solution with high temporal and spatial resolution. In recent years, many breakthroughs have been made in studying the nucleation, growth, etching, and self‐assembly mechanisms of nanomaterials.^[^ ^18^, ^19^, ^20^, ^21^, ^22^ ^]^ Specifically, welding in a solution environment does not require the application of additional force. In this study, we conducted an in situ investigation of the cold welding process using liquid cell TEM. Considering the excellent physicochemical properties and extensive application in electronics, biomedical, and plasmonics,^[^ ^23^, ^24^, ^25^ ^]^ noble metal Au nanorod with geometric anisotropy is chosen as the model system. The welding process of nanorods with different orientations was monitored and recorded in real‐time to elucidate the welding mechanism. High‐resolution TEM (HRTEM) images were captured to reveal the crystal structure of welded interfaces. Furthermore, theoretical calculations were conducted to unveil the underlying mechanism governing the welding of two nanorods through facet‐dependent surface diffusion and rearrangement.

## 2 Results and Discussion

### 2.1 Experimental Setup of In Situ Liquid Cell TEM

**Figure 1:**
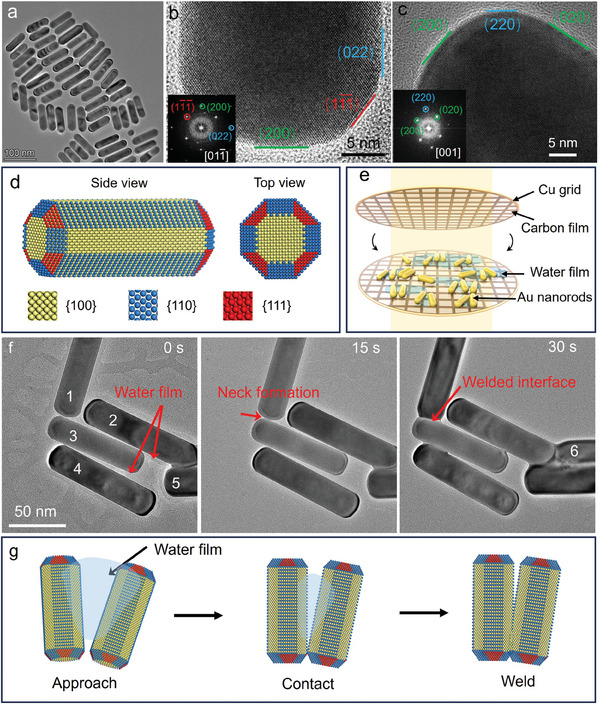
The cold welding of Au nanorods. a) TEM image shows the morphology of Au nanorods. b, c) HRTEM images show the facets at the tips and sides of the nanorods with different zone axes. d) Schematic illustrates the surface facets of Au nanorod. e) A schematic illustrates the setup of nanorods welding in the liquid cell. TEM frames from the in situ Movie S1 (Supporting Information) f) and schematics g) illustrate the welding process of nanorods.

**Figure** **1**a–d shows the morphology and the surface facets of Au nanorods viewed along different zone axes. These nanorods possess different facets at the surface, making them an ideal model system for elucidating the effects of lattice matching on the welding behavior of nanorods. The schematic of the experimental set‐up is depicted in Figure 1e, where two ultra‐thin carbon films supported by copper frames were employed to enclose the solution. This configuration enables us to capture HRTEM images with lattice resolution. The Au nanorods were synthesized via the polyol process, resulting in a distribution of diameters ranging from 10 to 30 nm and lengths between 80 and 100 nm. Before dropping them onto the copper frames, the capping agent at the Au nanorod's surface is carefully removed using the ion exchange (H^+^‐CTA^+^) method and redistribution in water (see the experimental details in the methods). The Zeta potential of washed Au nanorod's aqueous solution is ≈ 5 mV, indicating that the surfactant has been effectively removed.

### 2.2 The Cold Welding Process of Au Nanorods

Figure 1f,g illustrates the welding process of Au nanorods. Thin water films surrounding the nanorods could be identified through the contrast in TEM images (Figure 1f; Figure S1, Supporting Information). At first, the nanorods moved toward each other driven by water film‐induced capillary force. Upon contact, a narrow neck formed between the surfaces of the two nanorods at 15 s. Subsequently, the nanorods continue to rotate within the plane (with the zone axes of the nanorods remaining unchanged) to approach, and the atoms on the surface of the nanorods diffuse toward the high‐energy neck region due to the small curvature radius. Interestingly, different welding modes were observed depending on the relative position of the nanorods before welding. The “side‐to‐side” welding was carried out when the nanorods were close to parallel, like nanorods 2 and 3, 3 and 4. When the nanorods were close to vertical, they exhibited “top‐to‐side” welding mode, like nanorods 1 and 2.

To note that, the temperature rise induced by electron beam irradiation is no more than several Celsius which can be ignored (see more discussion in the methods). And the isolated Au nanorod is quite stable under a long time electron beam irradiation. As welding progressed, the Au nanorods maintained their crystalline structure, with no evidence of fusion or liquid‐like phases. Additionally, there were no morphological changes observed in the non‐welded parts of the nanorods with prolonged irradiation. Similar welding phenomena of Au nanorods were also observed at room temperature in the previous report.^[^ ^16^, ^26^ ^]^ Therefore, the welding process in our system can be classified as cold welding. To further eliminate the influence of electron beams on the welding of nanorods and clarify the role of water, controlled experiments in a vacuum under similar electron irradiation conditions were carried out (see more in Figures S2 and S3, Supporting Information). The results demonstrated the influence of electron beams on the welding mechanism of nanorods can be ignored, and the presence of water provides the driving force for the nanorods to approach each other, which can promote the occurrence of welding.

**Figure 2:**
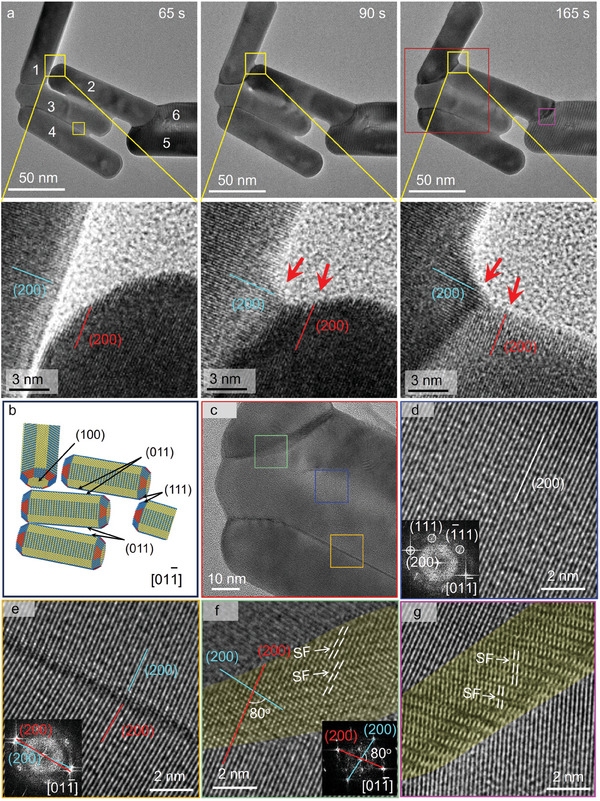
The details of Au nanorods welding process. a) TEM frames from the in situ Movie S1 (Supporting Information) show the welding process of nanorods with the same zone axis [01$\bar{1}$]. The corresponding enlarged image depicts the surface diffusion and rearrangement along the {200} facets to fill the gap between the nanorods. The red arrows point to the front edge of the welding interface, which can identify the {200} facets belonging to two nanorods. b) A schematic illustrates the view zone axis and surface facets of Au nanorods upon the welding. c) The HRTEM image of corresponding red squares in a) displays different welded interfaces between nanorods. d–g) The enlarged images of a corresponding colored square in c) and a) show the lattice‐resolved structures of the welded interfaces.

In order to reveal the welding mechanism at the nanoscale, TEM image sequences and corresponding enlarged images highlight the evolution of the welding interfaces (as shown in **Figure** **2**a). According to the HRTEM image (Figure S4, Supporting Information) and the schematic diagram (Figure 2b), nanorods 1 and 2 exhibit (002) facets at the contact surface before the welding. Upon contact, two {200} facets with an angle in the gap belonging to nanorods 1, and 2 could be identified, forming a welding interface between the nanorods. This suggests that the nanorods 1 and 2 undergo surface diffusion and rearrangement along their respective {200} facets to fill the gap during the welding process. The {100} facets cannot be observed in the TEM image due to the extinction of Au (face‐centered cubic structure). It should be noted that atoms diffuse along the {200} facets in the diameter direction of the nanorods, rather than along the contact surface of the nanorods. The nanorods 2, 3, and 4 exhibit (022) facets on the side surface before the welding which is different from the welding scenario of nanorods 1 and 2 (Figure S4, Supporting Information; Figure 2b). Interestingly, nanorods 2 and 3, and nanorods 3 and 4 were also welded together through the surface diffusion and rearrangement along the {200} facets (see more welding details in Figures S5 and S6, Supporting Information).

Although the above‐mentioned nanorods are all welded along the {200} facts, different welding interfaces can be distinguished from the contrast in Figure 2c, which suggests the different welding behaviors between nanorods. Noteworthy, the structure of the welded interface is crucial as it can potentially affect the mechanical properties, electronic transmission, and phonon vibration of the materials.^[^ ^27^, ^28^ ^]^ The misorientation angle between the nanorods 2 and 3 is as minute as 3° at the beginning of welding and gradually decreases as the nanorods approach during the welding process. They finally form the continuous {200} facets at the interface after welding (Figure 2c). If the nanorods are arranged in parallel and their top surfaces are approaching each other, the nanorods would welded in the “top‐to‐top” mode. Similarly, since they are well aligned, the nanorods are also welded through {200} facets, forming a continuous lattice at the welded interface (Figure S7, Supporting Information). However, when the angle between the nanorods increases to 5° such as in nanorods 3 and 4, low‐angle grain boundaries can be identified in the welded interface and cannot be eliminated through relaxation (Figure 2e).

For the welding between nanorods 1 and 2, and nanorods 1 and 3, the angles between the nanorods orientations are ≈80° before welding (as shown in Figure 2a). The welded interface in this scenario was quite different from the “side‐to‐side” welding of nanorods 2 and 3, and nanorods 3 and 4. The width of the welded interface, distinguishable by the TEM image contrast, was much larger as shown in Figure 2b–f. The red and blue lines with an angle of 80° represent the {200} facets belonging to each nanorod, indicating that the surface diffusion along their respective {200} facets formed a welded interface with defects such as stacking faults, without recrystallization into a typical face‐center cubic structure. In addition, if the nanorods undergo significant deformation during the welding process, regardless of the angle between their orientation, stacking faults would be formed at the welded interface due to the local stress (Figure 2g).

### 2.3 The Cold Welding of Au Nanorods with Different Zone Axes

**Figure 3:**
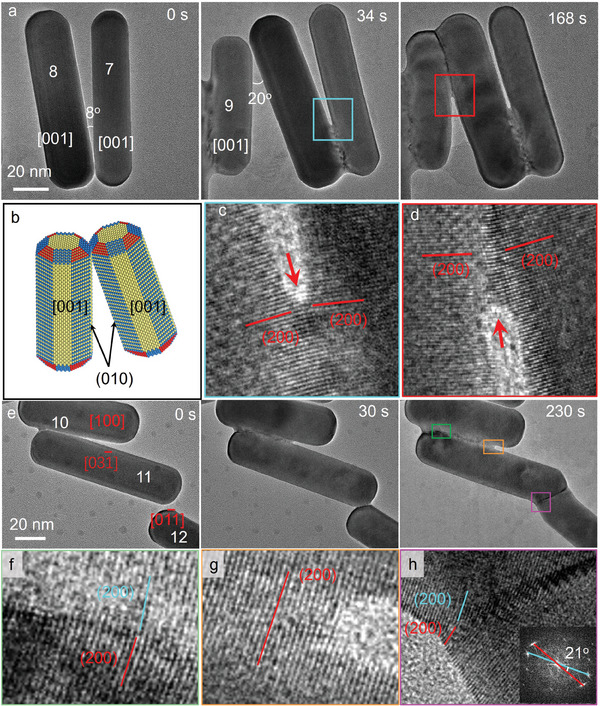
“Side‐to‐side” and “ tip‐to‐tip” cold welding of Au nanorods with different zone axes. a) TEM frames from the in situ Movie S2 (Supporting Information) show the welding process of nanorods with the [001] zone axis. b) A schematic illustrates the surface facets of Au nanorods upon the welding. c, d) The enlarged images of corresponding blue and red squares in a) show the lattice‐resolved structure of the welded interface. e) TEM frames from the in situ Movie S3 (Supporting Information) show the welding process of nanorods with different zone axes. f–h) The enlarged images of the corresponding green, orange, and purple squares in e) show the lattice‐resolved details of the welded interface. The red and blue lines indicate the {200} facets belong to different nanorods.

As discussed above, the nanorods with the zone axis [01$\bar{1}$] are always welded through surface diffusion and rearrangement along the {200} facets. In order to verify the universality of the facet‐dependent surface diffusion, the welding of nanorods with different zone axes is shown in **Figure** **3**. Figure 3a illustrates the “side‐to‐side” welding of nanorods with the zone axes of [001] Movie S2, Supporting Information). Before coming into contact, the two nanorods exhibit (020) facets at their surface (Figure 3b; Figure S8, Supporting Information). Similarly, the nanorods also demonstrated surface diffusion and rearrangement along the {200} facets to fill the gap (Figure 3c,d). Since the angle between nanorods is larger than 5°, grain boundaries can be found in the welded interface, similar to the welding of nanorods 3 and 4 in Figure 1.

Besides, nanorods 10 and 11 with different zone axes (Figure S9, Supporting Information) also experienced surface diffusion and rearrangement along the {200} facets during the welding. Initially, they formed low‐angle grain boundaries, and then a continuous lattice at the interface when the angle between two nanorods approaches zero (Figure 3f,g). If the nanorods are welded in a “ tip‐to‐tip” mode, such as the nanorods 11 and 12 shown in Figure 3e, in addition to the welding along the {200} facets (Figure 3h), the nanorods underwent significant deformation. The angle between the (200) facets belonging to each respective nanorod increased from 12° to 21°. Due to misalignment of the {200} facets and local stresses caused by nanorods deformation, many defects were formed at the welded interface which is similar to the welding of nanorods 2, 5, and 6 in Figure 1. The results of these different welding modes indicate that the welding of the Au nanorods along the {200} facets is ubiquitous and independent of the contact surface facets. While the morphology and structure of the welded interface are determined by the orientation angle of the nanorods.

It's worth noting that, this welding phenomenon differs significantly from the previously reported oriented‐attachment (OA) process which is considered as an important mechanism of nanowelding and growth of nanocrystals in many systems.^[^ ^3^, ^29^ ^]^ Usually, two nanoparticles with high mobility approach each other and rotate continuously until their interfaces share a common crystallographic orientation and then jump to contact. During the OA process, nanoparticles always merge into a single crystal through a common lattice facet or develop twin structures in the presence of an initial misalignment upon attachment.^[^ ^29^, ^30^, ^31^ ^]^ While nanorods in our experiments do not rotate to achieve the same facets at the surface before welding due to the low mobility of nanorods. The driving force for welding is the decrease of surface energy associated with forming a new structure. Therefore, the nanorods fill the gap through surface diffusion and rearrangement along specific facets, independent of the contact facets. The structure of the welding interface is related to the initial orientation of the nanorods before welding. The nanorods can only form continuous facets through welding when the angle between their orientation is less than 3°. When the angle is larger, the defects at the welding interface such as small angle grain boundaries and 3, cannot be eliminated.

### 2.4 Theoretical Calculations

**Figure 4:**
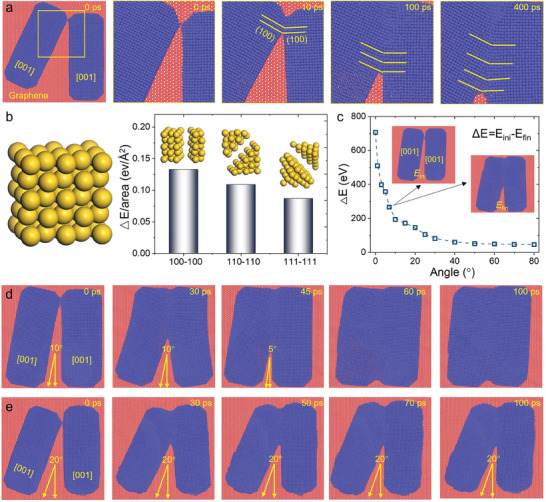
MD and DFT calculations for the cold welding of Au nanorods. a) The surface diffusion and rearrangement of atoms along the {100} facet during the cold welding process. b) Comparison of the dissociation energies of {100}, {110}, and {111} facets. c) The energy is released during the cold welding of two Au nanorods with varying included angles. d, e) Atomic snapshots in the cold welding process with small and large initial angles between Au nanorods.

To demonstrate the atomic behavior and reveal the mechanism in the nanorod welding process, molecular dynamics (MD) simulations, and density functional theory (DFT) calculations are performed. **Figure** **4**a shows the enlarged atomic snapshots at the welded interface in a time series. It can be found that the connecting of two nanorods is via the surface diffusion and rearrangement of atoms along the (100) facets, consistent with the experiments (Figures 2 and 3). To elucidate why nanorods are always welded along the {100} facets, DFT is used to calculate the dissociation energies, *E*_dis_, of {100}, {110}, and {111} facets by separating an Au crystal along these facets, respectively, and comparing the energy variations (Figure 4b). The dissociation energies of {100} facet are 0.133 eV Å^−2^, higher than 0.109 eV Å^−2^ of {110} and 0.087 eV Å^−2^ of {111} facets. This implies that the coalescence of two {100} surfaces results in the maximum energy release, thereby rendering the welding along the {100} facet during the welding process that is thermodynamically favorable.

To further discover the effect of the initial angle between two nanorods on the welding process and atomic structure, MD simulations for the angles varying from 0 to 80° are carried out, from which the energy releases after the welding are obtained (Figure 4c). A monotonically declining trend of the energy is shown, indicating that a large angle decreases the driving force for the nanorods to weld. The higher driving force makes nanorods with small angles well welded with continuous lattice or low‐angle grain boundaries (Figure 2d,e). However, as a driving force in large‐angle welding is too weak, the welded interface has a much larger area and disordered structure (Figure 2f,g). We also compared the movement of nanorods in the welding process for different initial angles (Figure 4d,e). When the angle between two nanorods is small (10°), low‐angle grain boundaries are formed at the welded interface. As the welding progresses, nanorods rotate to approach and the angle between the nanorods gradually decreases. When the angle is small enough, the nanorods minimize defects in the welded interface through atomic relaxation (Figure 4d), corresponding to the welding between nanorods 2 and 3 (Figure 2a), and 10 and 11 (Figure 3e). As a contrast, nanorods with 20° angle welding present a stable angle during the welding process, and even the welding area stops to increase because they cannot rotate to get closer to each other (Figure 4e). The rotation to each other for a small initial angle is owing to the higher welding driving force (Figure 4c). While for a large initial angle, the driving force is not enough to drive the rotation, resulting in partial welding.

## 3 Conclusion

In this study, we demonstrate the cold welding of the Au nanorods without the requirement of external load or induced heat. Various welding methods of nanorods are studied in detail, and it is found that the nanorods do not need to rotate to align the surface facets before welding. In situ, lattice‐resolved characterization of the welding process and the MD simulation reveal that the welding of Au nanorods always progresses through the surface diffusion and rearrangement of atoms along the {100} facets, regardless of the nanorods zone axes and the contact surface facets. DFT calculations indicate that the maximum energy released by the coalescence of the {100} facets is the fundamental reason for the facets‐dependent surface diffusion and rearrangement in the welding process. The effects of misalignment of {200} facets and local deformation on the welded interface structure are fully revealed. Our findings clarify the fundamental mechanisms governing the cold welding process of metal nanomaterials and present a promising approach for the next‐generation interconnects and self‐healing of metallic nanostructures.

## 4 Experimental Section

### In Situ Liquid Cell Experimental Setup

The Au nanorods were synthesized via the polyol process and washed with deionized water twice to remove the redundant surfactant cetyltrimethylammonium bromide (CTAB).^[^ ^32^ ^]^ Ion exchange (H^+^‐CTA^+^) method is used to remove CTAB molecules from the surface of the Au nanorods. First, the Au nanorod's solution was mixed with 50 mm hydrobromic acid (vol: 1:1) and stirred for 5 min under the argon atmosphere at room temperature. Then the mixed solution was centrifuged at 10 000 rpm for 10 min to remove the supernatant and Au nanorods were redispersed in deionized water.

The liquid cell was set up using a method similar to that reported in the previous study.^[^ ^22^ ^]^ First, a droplet (2.5 µL) of Au nanorods solution (50 µg mL^−1^) was drop‐casted onto an ultra‐thin carbon film TEM grid. Then the wet grid with another grid was covered. After the liquid cell was assembled, it was placed for more than 4 h to evaporate excess solution before loading into the TEM for imaging.

### TEM Characterization

The TEM Characterization and in situ experiments were carried out using FEI ThemIS with a Cs‐corrector operated at 300 kV. An incident electron dose rate of 1000–1600 e^−^ Å^−2^⋅s^−1^ is maintained for the study.

### Electron Beam Induced Temperature Increases

The heating effect of electron beam irradiation on the aqueous solutions was first considered. In the experiments, the beam current *I* ≈7.17 nA, 8.27 nA with the illuminated area of 300 and 375 nm in radius. According to Joseph M. Grogan et al., ^[^ ^33^ ^]^ for the beam current < 10 nm, with the acceleration voltage of 300 keV, the maximum temperature rise is less than 4 Celsius. Considering that the water film gradually disappears during the welding process, Li's paper was referred to calculate the temperature rise of gold nanorods caused by the electron beam in the absence of water.^[^ ^31^ ^]^ The thermal conductivity of Au ≈300 W·m^−1^·K^−1^, and the temperature increase was estimated to be less than 1 Celsius.

### Theoretical Study

Density functional theory (DFT) calculations were carried out based on the Vienna Ab initio Simulation Package (VASP).^[^ ^34^, ^35^ ^]^ PBE‐GGA methods were used to describe the pseudopotentials and exchange‐correlation functionals.^[^ ^36^ ^]^ The cutoff of a plane wave was 520 eV with self‐consistent field (SCF) energy convergence of 1 × 10^−8^ eV. Monkhorst–Pack meshes with a k‐spacing value of 0.020 in Vaspkit were set,^[^ ^37^, ^38^ ^]^ which resulted in a 2 × 2 × 2 k‐mesh. In the calculations of dissociation energies, an Au crystal of 20.4 Å × 20.4 Å × 20.4 Å with 500 Au atoms was adopted to cut different faces out (see Figure S10a, Supporting Information). The dissociation energies, *E*_dis_, can be expressed as,

(1) $$E_{dis}=\left(E_{cry}-E_{part1}-E_{part2}\right)/A_{surface}$$

where *E*_cry_ is the free energy of the Au crystal before division, *E*_part1_ and *E*_part2_ are the free energies of the two parts from dividing the crystal, and *A*_surface_ indicates the surface area that connects the two parts. When dividing the crystal along {100}, {110}, and {111} facets, the obtained *E*_dis_ indicates the dissociation energy of the corresponding facets. Molecular dynamics (MD) simulations were performed based on the Large‐Scale Atomic/Molecular Massively Parallel Simulator (LAMMPS). Considering the interactions between Au nanorod and the carbon film could affect the welding process, graphene as the substrate was used in the simulations to simulate to represent the effect of carbon film on the nanorod welding. The force field among Au atoms is from the embedded atom method (EAM),^[^ ^39^ ^]^ and CH airebo potential is applied for graphene. Lennard‐Jones potential between Au and C atoms was used.^[^ ^40^ ^]^ All the MD simulations were a time step of 1 fs and a temperature of 300 K. The atomic models and the sizes for MD simulations are shown in Figure S10b (Supporting Information).

## Conflict of Interest

The authors declare no conflict of interest.

## Author Contributions

W.W., T.X., H.‐M. Z and L.‐T. S. conceived and designed the experiments. W.W. performed the experiments; D.‐X. S. developed the simulations. F.‐J. M., S.‐F. F., and S.‐B. C. took part in the discussion and data analysis; R. C synthesized the samples; L.‐T. S. supervised the project and revised the paper with C. ‐X. S., H.‐M. Z., and T. X. The manuscript was written through contributions of all authors. All authors have given approval to the final version of the manuscript.

## Supporting information



Supporting Information

Supplemental Movie 1

Supplemental Movie 2

Supplemental Movie 3

## Data Availability

The research data that support the findings of this study are available from the corresponding authors upon request.
