# Supplementary material for: Facet‐Dependent Cold Welding of Au Nanorods Revealed by Liquid Cell Transmission Electron Microscopy
Source: Adv Sci (Weinh). 2025 Jan 31;12(12):2412779. doi: 10.1002/advs.202412779 (PMC11948076; doi:10.1002/advs.202412779)
Supplement: Supplementary file 1 — Supporting Information [file ADVS-12-2412779-s003.docx]

Supporting Information

Facet-dependent cold welding of Au nanorods revealed by liquid cell transmission electron microscopy

Wen Wang, Dongxing Song^*^, Fangjie Meng, Sufeng Fan, Ran Cai, Shaobo Cheng, Chongxin Shan, Tao Xu^*^, Haimei Zheng^*^, Litao Sun^*^

**Supplementary Materials:**

Supplementary Figures and Figure Captions S1 to S10

Supplementary Videos Captions 1 to 3

References

**Other Supplementary Materials include the following:**

Supplementary Videos 1 to 3

**Video Captions**

**Supplementary Video 1. The cold welding process of Au nanorods with the zone axis [01**$\overline{\boldsymbol{1}}$**].**

**Supplementary Video 2.** **The“ side-to-side” cold welding of Au nanorods with the zone axis [001].**

**Supplementary Video 3.** **The cold welding process of Au nanorods with different zone axes.**


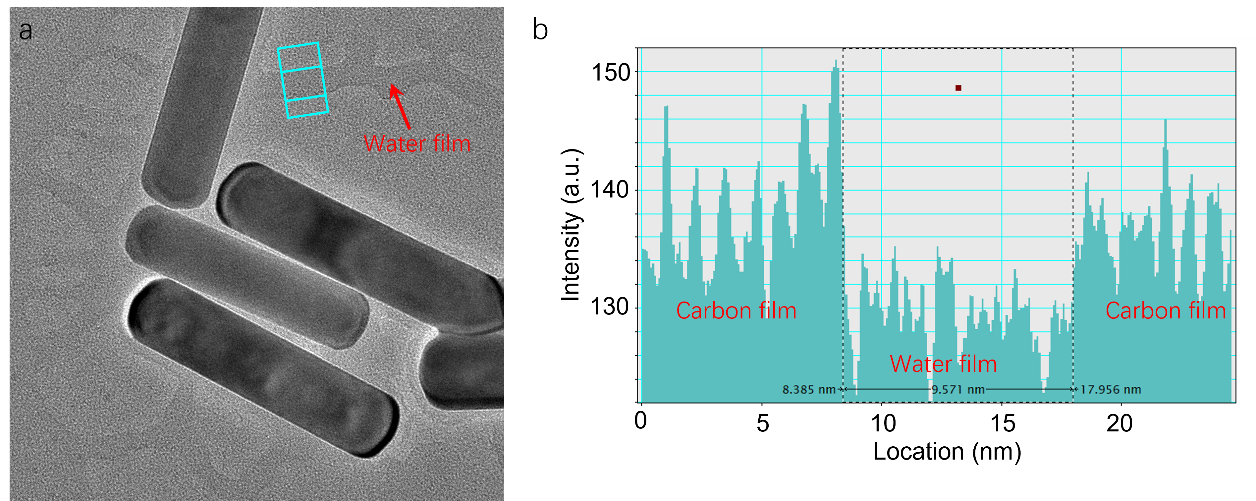


**Figure** S1. Characterization of the water film surrounding the nanorods. a. TEM image of the Au nanorods surrounded by water film. b. Intensity profile of the selected area shown in a. The thin water films exhibit lower intensity than a carbon film.

In order to clarify the role of water solution and influence of electron irradiation in the welding, we conducted controlled experiments in vacuum under similar electron irradiation conditions. The Au nanorods solution was dropped onto the TEM grid and was put into the TEM after drying. When the nanorods are separated at a distance, no welding occurs after 200 s irradiation since the nanorods are pined to the substrate and difficult to move (Figure. S2). Afterwards, we investigated the behavior of nanorods at different distances and electron beam dose rates. The nanorods still did not approach each other or weld together even though we increased the dose rate (Figure. S2 b, c and d). However, if the nanorods are well aligned and placed close enough to reach the short-range interaction range between them, welding could occur after prolonged irradiation (Figure. S3). High-resolution TEM images show that the nanorods were welded together through the {200} facets. This welding mechanism is similar with what we observed in the solution, but the speed is slower at least an order of magnitude. Therefore the influence of electron beam on the welding mechanism of nanorods can be ignored, and the presence of water provides the driving force for the nanorods to approach each other, which can promote the occurrence of welding.


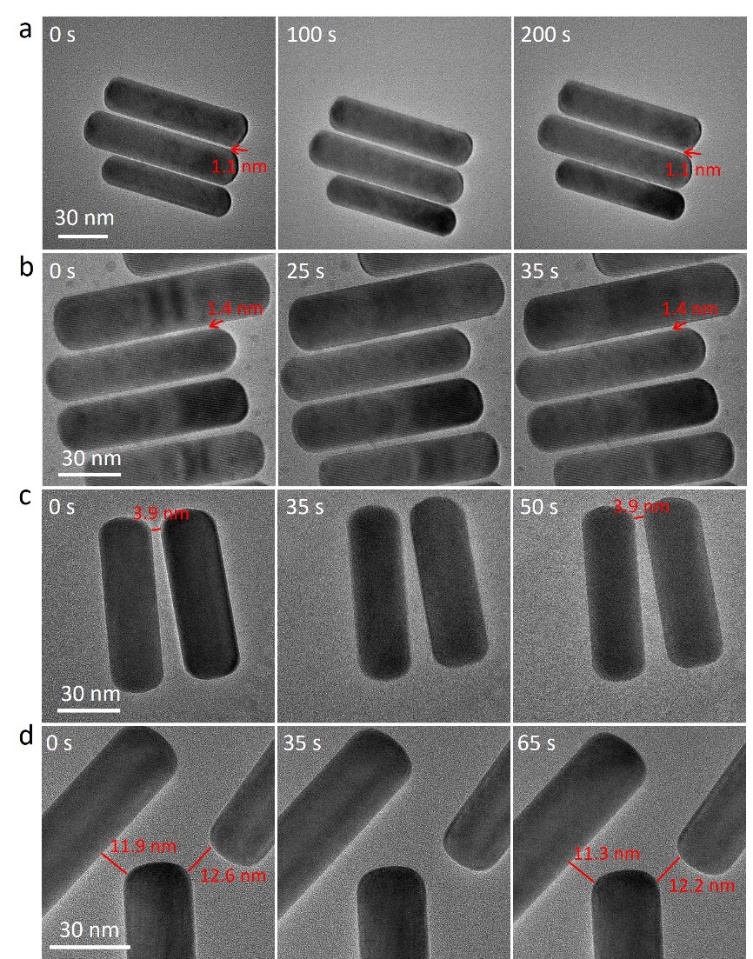


**Figure** S2. The separated gold nanorods did not undergo welding in vacuum with different electron beam dose rates irradiation. a. Dose rate: 1526 e^-^/Å^2^⋅s, b. Dose rate: 2440 e^-^/Å^2^⋅s, c. Dose rate: 2440 e^-^/Å^2^⋅s, d. Dose rate: 3200 e^-^/Å^2^⋅s.


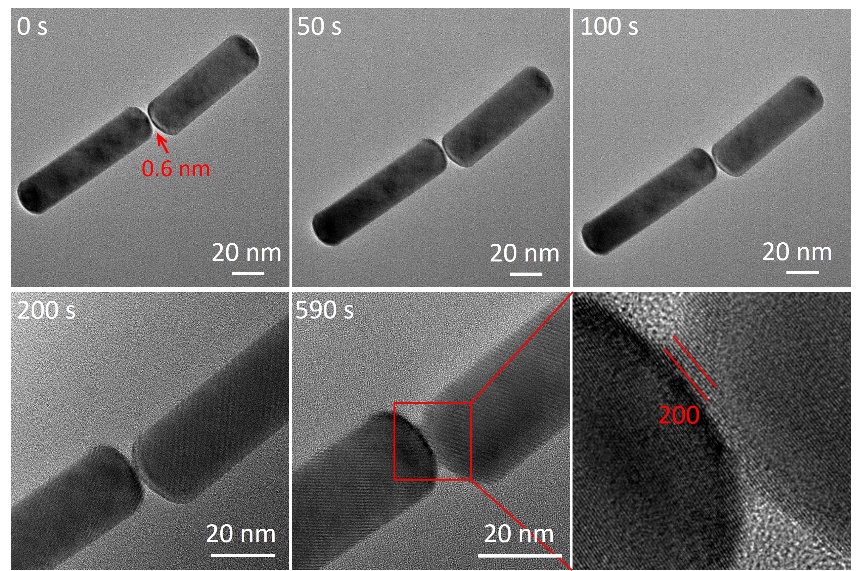


**Figure** S3. The welding of nanorods with a separation distance of 0.6 nm


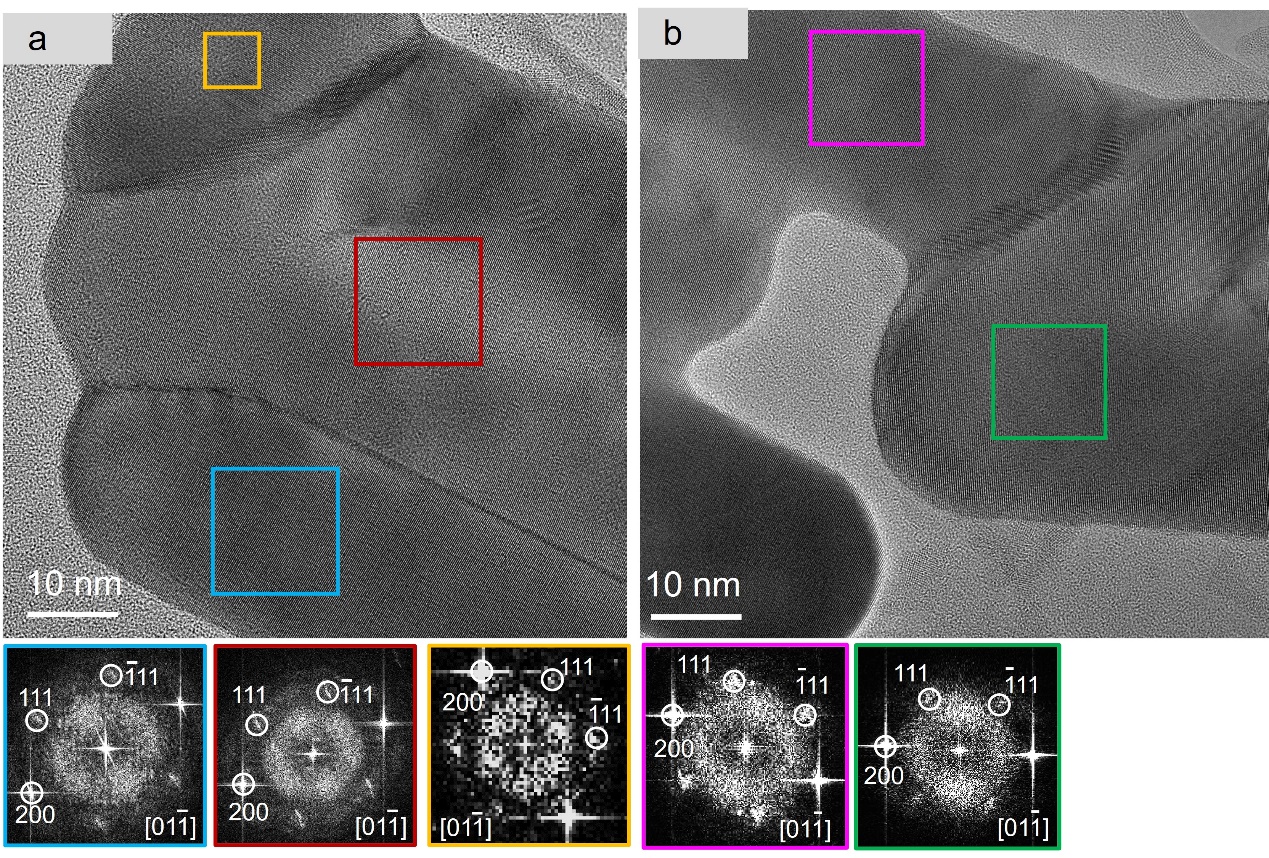


**Figure** S4. The HRTEM image of naorods in movie S1. The FFT of the different nanorods clearly show the same [01$\overline{1}$] zone axis.


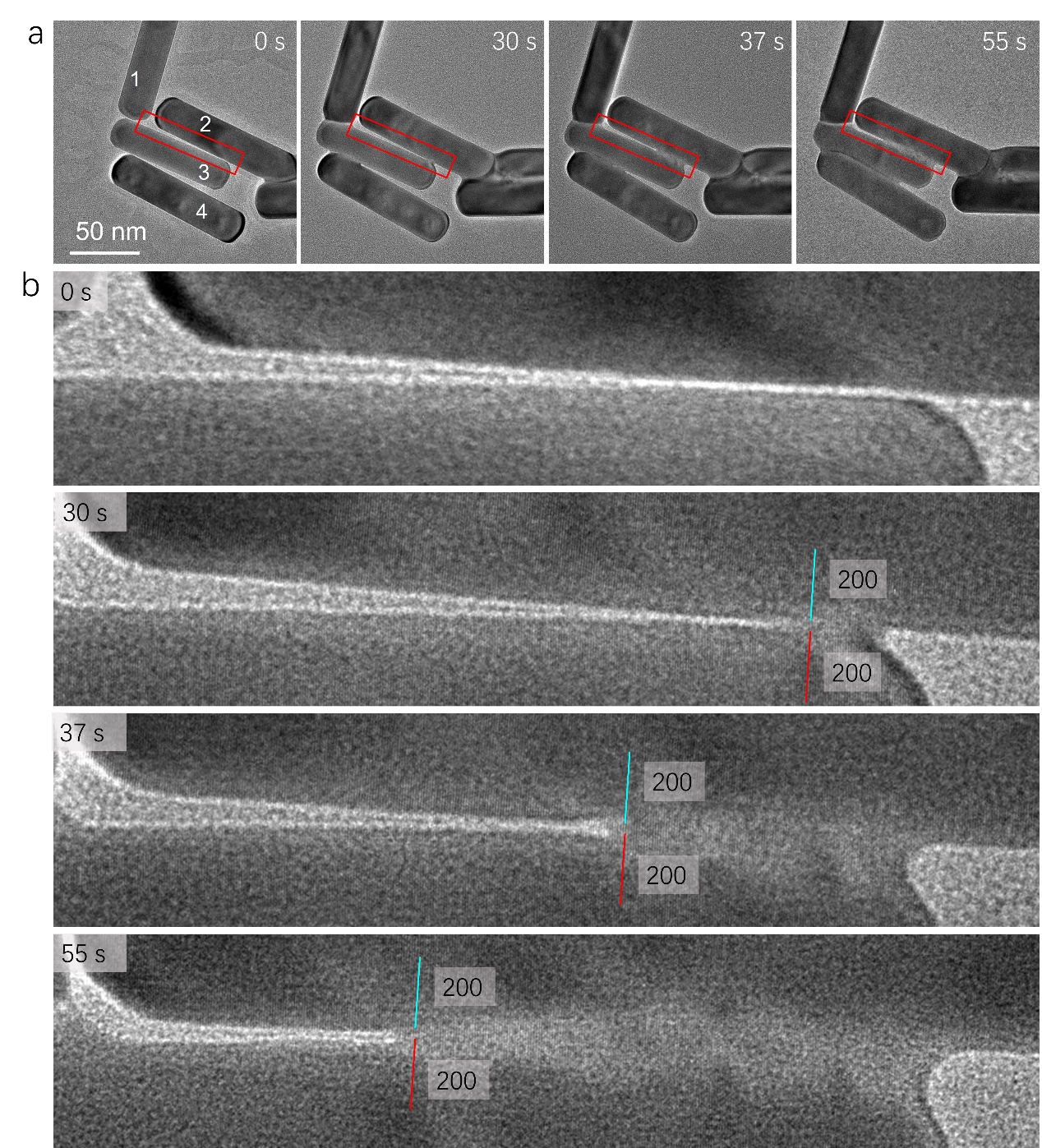


**Figure** S5. The welding process of nanorods 2 and 3. a. TEM image sequences show the welding process of the nanorods 2 and 3. b. The enlarged images of corresponding red rectangular display that the nanorods were welded together through the surface diffusion and rearrangement along the {200} facets to fill the gap.


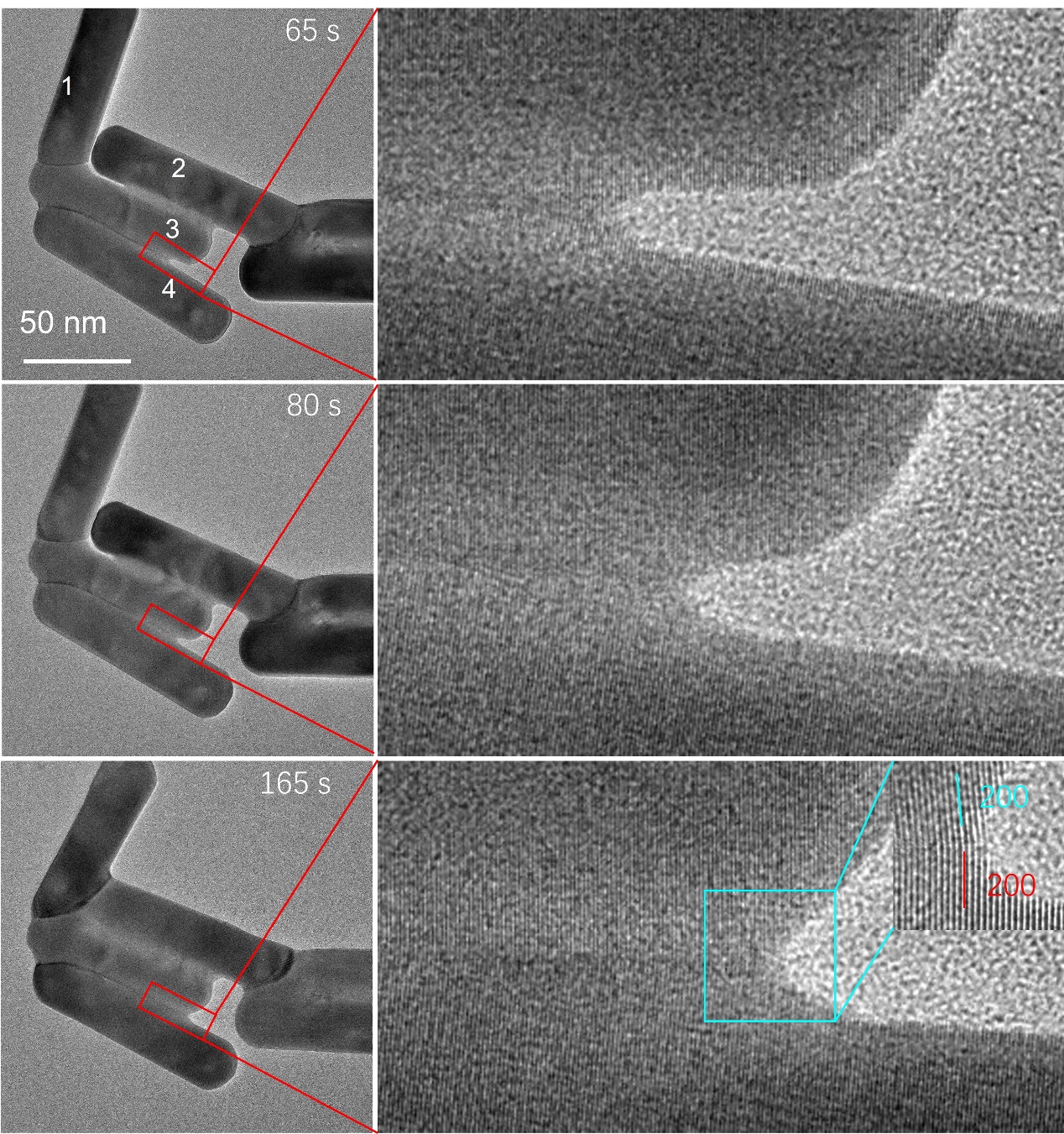


**Figure** S6. TEM image sequences and the corresponding enlarged images show the welding of the nanorods 3 and 4 through the surface diffusion and rearrangement along the {200} facets.


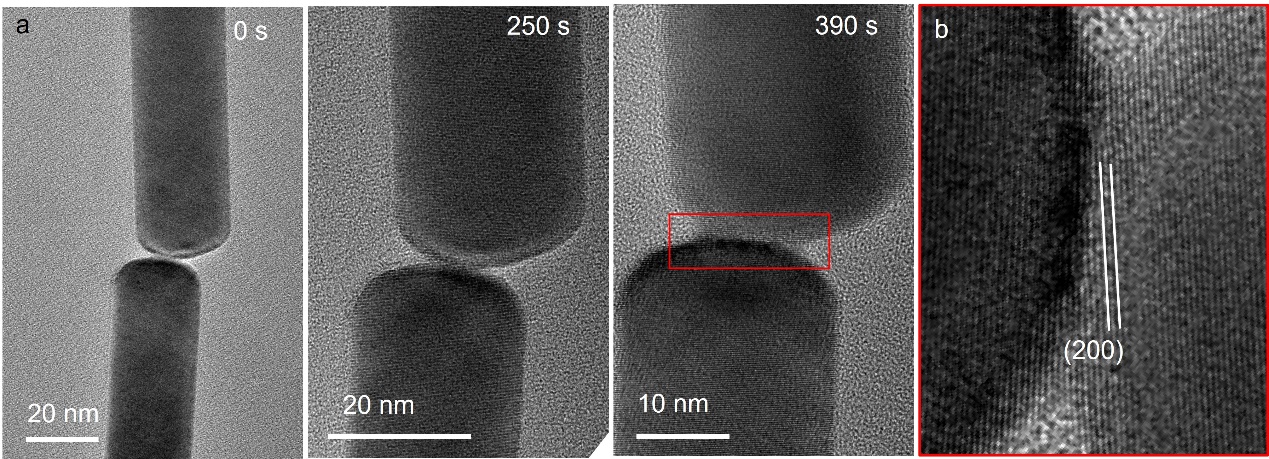


**Figure** S7. Top-to-top welding of Au nanorods. a. TEM frames show the top-to-top welding process of nanorods with same orientation. b. The enlarged images of corresponding red square in (a) show the lattice-resolved details of the welding interface. The orientation of the nanorods before welding is similar, forming a continuous lattice at the welded interface.


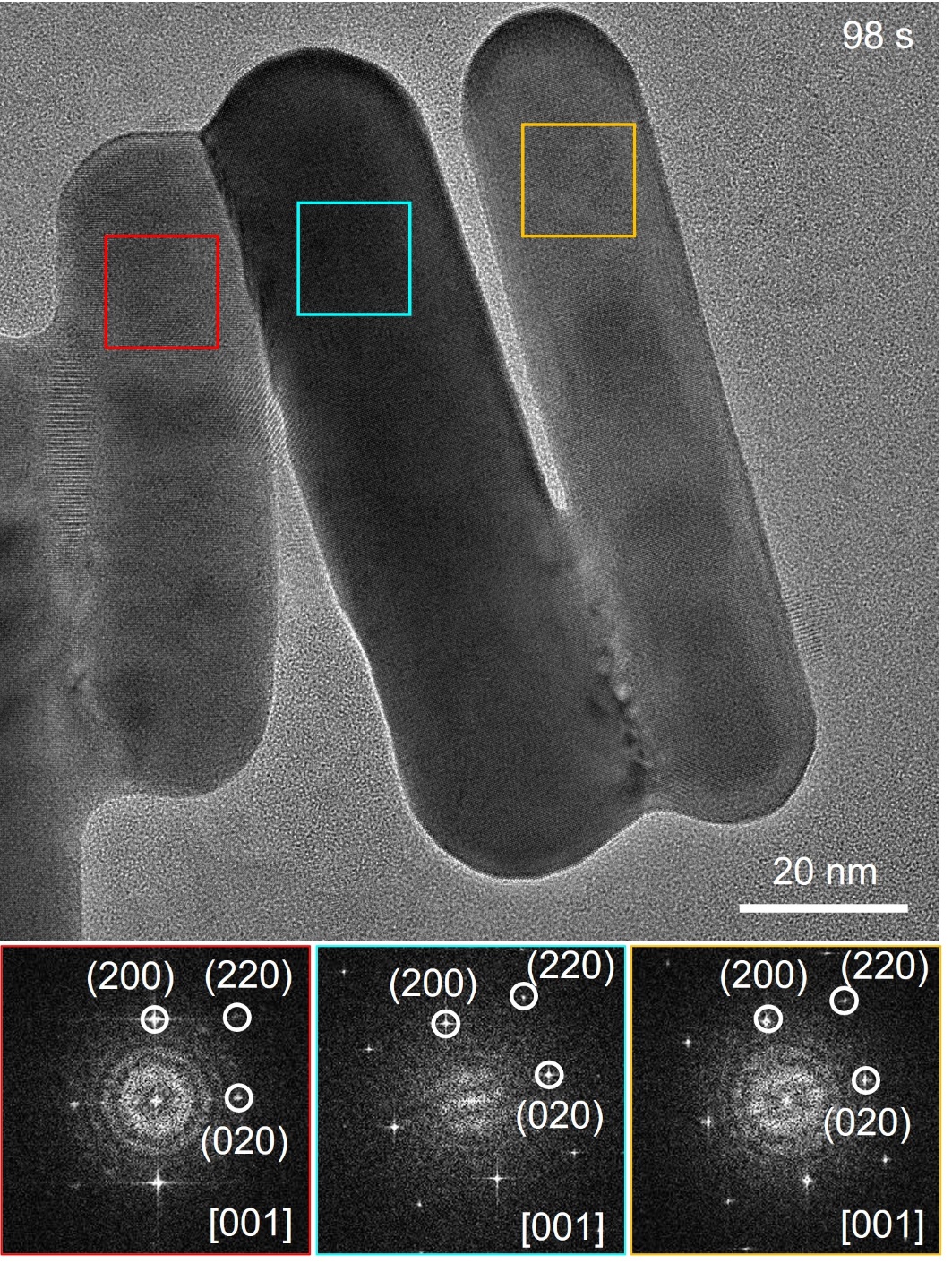


**Figure** S8. The HRTEM image of naorods in movie S2. The FFT of the different nanorods clearly show the same [001] zone axis.


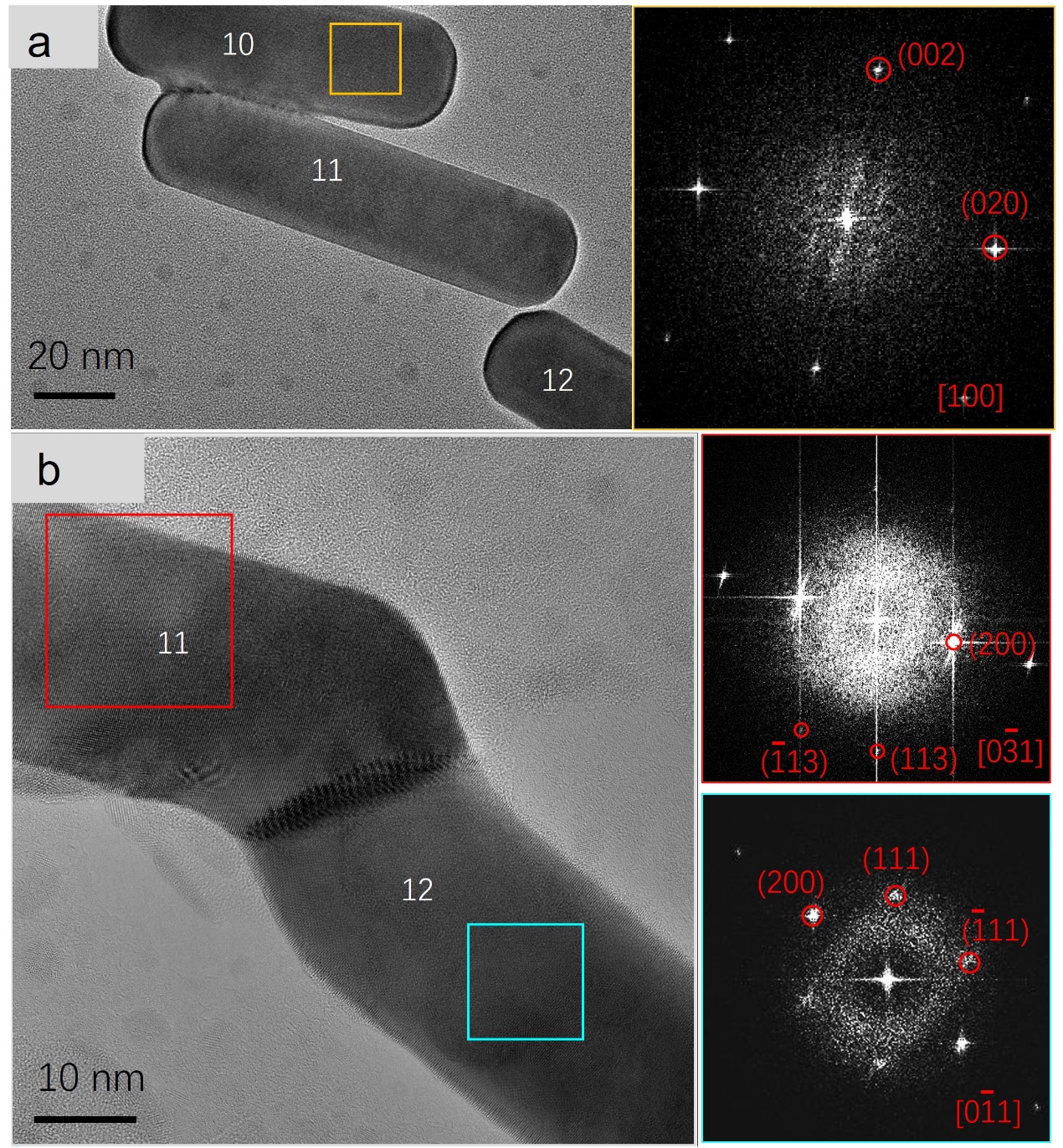


**Figure** S9. The HRTEM image and the corresponding FFT images of naorods in movie S3.


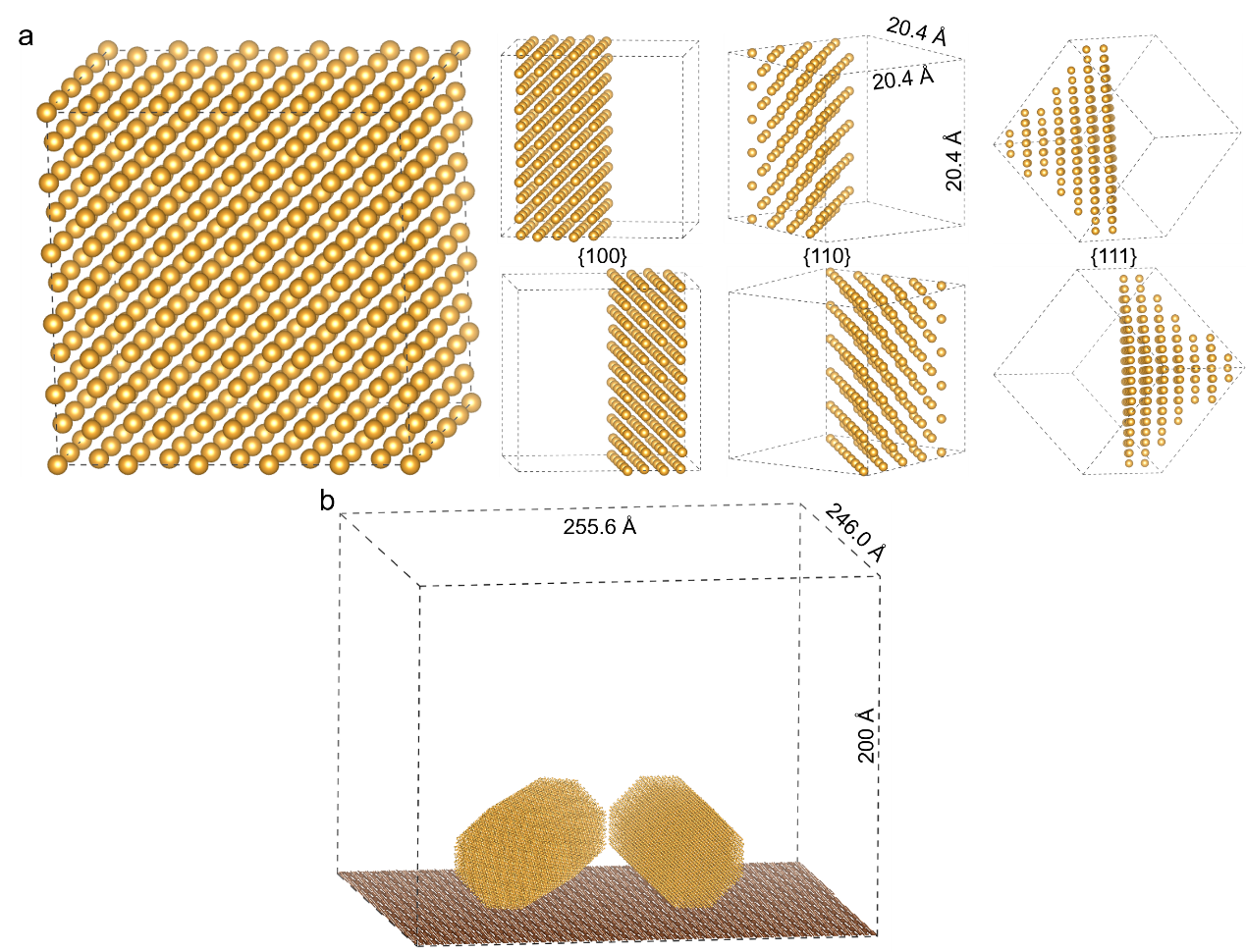


**Figure** S10. Atomic models and the sizes. a. DFT calculations. b. MD simulations.
